# Supplementary material for: Are carbon emissions trading and green financial instruments synergistic? -Comprehensive quantitative research based on content analysis
Source: PLoS One. 2024 Mar 7;19(3):e0298601. doi: 10.1371/journal.pone.0298601 (PMC10919601; doi:10.1371/journal.pone.0298601)
Supplement: S1 Data — (ZIP) [file pone.0298601.s001.zip › Data/地级市Z_F工作报告.zip!/╕╩╦α/╢¿╬≈╩╨/└┤╫╘╣1⁄2╓┌║┼í╛┬φ┐╦╩2╛▌═°í┐_2013.docx]

各位代表：

　　现在，我代表市人民政府，向大会作工作报告，请予审议，并请各位政协委员和列席会议人员提出意见。

　　2012年工作回顾

　　2012年，市政府在省委、省政府和市委的坚强领导下,在市人大、市政协的监督支持下，认真贯彻党的十八大和省第十二次党代会、市第三次党代会精神，紧盯与全省全国同步进入全面小康社会宏伟目标，抢抓机遇，攻坚克难，狠抓落实，圆满完成了市三届人大一次会议确定的各项目标任务，开创了经济转型跨越发展、人民生活持续改善、社会保持和谐稳定的新局面。预计全市完成生产总值215亿元，增长13%。固定资产投资395亿元，增长45%。社会消费品零售总额73亿元，增长17%。三次产业结构调整为27∶29∶44，二产占比首次超过一产。大口径财政收入23.2亿元，公共财政预算收入13.1亿元，分别净增5.4亿元、3.1亿元，分别增长30.7%、31.1%。公共财政预算支出148.7亿元，净增31亿元，增长26.3%。城镇居民人均可支配收入14281元，增长16.2%。农民人均纯收入3600元，增长17.1%。金融机构存贷款余额分别为410.1亿元和239.4亿元，分别增长26.6%和33.8%，贷存比58.4%，较年初提高3.2个百分点。人口自然增长率6.44‰，城镇登记失业率3.71%，居民消费价格指数102.8，节能减排任务全面完成。

　　一年来，我们主要抓了以下工作：

　　（一）致力扩大投资规模，不断加强项目工作。始终把扩大投资作为拉动经济增长的重要抓手，坚持争取国家投资与招商引资并举、基础设施建设与产业开发并重，以项目数量扩大经济总量，以投资结构优化产业结构。落实项目前期经费5900万元，编制马铃薯、中医药两个千亿元和畜草、蔬菜两个500亿元等专项规划32项，谋划项目2055个、总投资1.58万亿元，其中围绕全省“3341”项目工程，谋划项目1068个、总投资4562亿元。争取国家政策性资金65.4亿元，增长23%。实施500万元以上项目1311个，完成投资346.8亿元，其中亿元以上项目155个，完成投资134.3亿元。渭河源区生态保护与综合治理规划通过国家发改委组织的现场评估，临渭高速、通渭马营至陇西云田二级公路开工建设，定临、文殪二级公路基本建成。坚持大招商、招大商，执行招商引资项目274个，到位资金182.5亿元，增长111.2%，其中引进总投资20亿元的甘肃招金贵金属冶炼等亿元以上项目88个，到位资金97.3亿元,增长168.8%。第十八届“兰洽会”签约重点项目总投资505亿元，是前5年总和的1.3倍，已开工项目完成投资82.7亿元。争取设立了总规模2.5亿元的“甘肃生物医药产业创业投资基金”，重点扶持中医药、马铃薯产业发展。“5·10”岷县灾后恢复重建规划全面实施，到位资金16.82亿元，完成投资9.24亿元，灾民建房任务全面完成。全市项目工作在城市规划建设、工业发展和特色优势产业开发等方面取得的显著成效，赢得了省委、省政府和全省新建项目观摩会议的高度评价。

　　（二）致力提升一产水平，加快发展现代农业。坚持一产抓提升，大力发展马铃薯、中医药、畜草等特色优势产业。“中国薯都”建设依托产业园及良种繁育供应、标准化种植、精深加工和现代物流加快推进，并得到农业部确认和支持。生产马铃薯脱毒原原种5.1亿粒，建立原种扩繁基地4.5万亩、一二级良种基地160万亩、标准化种植基地268万亩，马铃薯种植面积326万亩、总产量498万吨。“中国药都”建设依托产业园及标准化种植、多层次加工和规模化交易加速推进。建立中药材优质种子种苗繁育基地6.4万亩、标准化种植基地51万亩，中药材种植面积108万亩、总产量22万吨，较上年增加5.3万吨。畜草产业发展势头强劲。投入财政扶持资金6000万元，新建规模养殖场233个，发展规模养殖户1万户，引进广德福丰等大型养殖龙头企业27户。全市畜禽规模养殖比重提高到35%，畜禽养殖总量达到1800万头（只），较上年增长44%。马铃薯、中医药、畜草产业总产值分别达到78.5亿元、160亿元和56亿元，“三足鼎立”发展局面开始形成。设施农业、旱作高效农业跟进发展，新建日光温室1248座、塑料大棚8700亩，蔬菜种植面积扩大到55万亩，较上年增加12.4万亩，推广全膜双垄沟播种植360万亩，较上年增加88万亩。增产粮食13.8万吨，总产量达到137.4万吨，实现了“五连增”。通渭、安定、临洮荣获全国粮食生产先进县（区）。培训城乡劳动力21.4万人，输转67.8万人次，实现劳务收入74.9亿元。引导组建农民专业合作社464个，筛选认定市级农业产业化重点龙头企业53户，流转土地55万亩，农业组织化、集约化和产业化程度不断提高。

　　（三）致力扩充二产总量，推动工业提质增效。坚持二产抓扩充，深入推进工业发展十大行动计划，实施华能华家岭风电一期等千万元以上工业项目216个。完成工业固定资产投资98.5亿元，增长35.6%。安排财政专项资金1.56亿元，扶持工业企业发展壮大，新增规模以上工业企业30户，累计达到80户，规模以上工业增加值增长23.5%。不断理顺经济开发区管理体制，投资10.4亿元加强基础设施建设，不断提升承载功能，经济开发区和工业集中区新入驻企业89户，累计达到904户，工业增加值占到全市工业增加值75%左右。马铃薯精淀粉及其制品加工能力达到40万吨，加工产品已向多用途变性淀粉、淀粉糖及壁纸胶、无矾方便粉丝等高端产品迈进。新增中医药加工企业34户，累计达到130户，带动中药材加工能力发展到10万吨。陇西经济开发区循环化改造试点进展顺利，定西节水型工农业复合循环经济模式列为全省十大循环经济典型案例予以推广。规范和扶持建筑业发展，新增资质以上建筑及房地产企业39户，累计达到129户，与工业共促二产加快发展。全市二产增加值增长20.9%，其中工业增加值增长19.7%。工业对财政贡献率达到22%。

　　（四）致力促进三产转型，不断增强发展活力。坚持三产抓转型，制定服务业发展规划，扶持引导生产性服务业加快发展，生活性服务业转型提高。大力发展文化旅游产业，成功举办首届中国·陇西李氏文化旅游节、漳县全国攀岩精英赛等大型节会，启动建设临洮马家窑、陇西李氏、岷县洮砚、通渭书画等文化产业园，完成投资4.38亿元。推动文化旅游产业加快融合发展，文化产业增加值、旅游综合收入分别增长26%和29.7%。高度重视品牌创建和高端市场建设，“定西马铃薯”被国家工商总局认定为中国驰名商标，飞天牌粉丝等7个产品荣获甘肃名牌称号，定西马铃薯批发市场升格为国家级市场并列为省部共建项目，安定区被中国证监会确定为陇薯期货交割基准地，中国·文峰药材交易城年交易额突破80亿元。加强金融服务体系建设，争取甘肃银行在定西设立了办事处、兰州银行在陇西设立了支行，新发展小额贷款公司8户、担保公司5户，累计分别达到19户。农村金融服务创新和综合改革试点深入推进，农村“三权”抵押贷款试点初见成效。保险业稳定发展。农产品电子交易等新型业态紧贴特色优势产业快速发展。新增生产性服务企业769户，累计达到3000多户。新增限额以上批零企业、住宿餐饮企业13户，累计达到66户。第三产业增加值增长12.8%。

　　（五）致力统筹城乡发展，加强基础设施建设。加大城乡统筹力度，积极推进户籍制度改革，引导农村人口向城镇有序转移，城镇化率达到26%左右。坚持高起点规划、高标准建设，各县城新一轮总体规划修编基本完成，市区和县城控制性详规编制率分别达到95%和85%。实施各类城建项目263个，完成投资99.8亿元，增长34%。市区实施项目89个，完成投资42.4亿元，增长10.7%。新城区集中供热等23个项目全面建成，市行政中心、薯都大道等37个项目进展顺利，新城区 “三年打基础、五年成规模”阶段性建设目标如期实现。旧城区供热管网改造等7个项目全面完成，南大街、气象新村等22个项目进展顺利。各县城和重点小城镇实施项目174个，完成投资57.4亿元，增长59%。陇西县省级新农村建设试点和安定区巉口等6个市级新农村建设试点镇顺利通过省市验收，新农村建设试点圆满结束。投资9.8亿元，实施交通项目172个，等级公路基本实现乡镇全覆盖，行政村通沥青或水泥路率达到45.5%。投资19.2亿元，实施重点水利工程43个，新增堤防68公里、节水及有效灌溉面积13.3万亩，新解决了42万农村人口饮水不安全问题。引洮一期定西支渠和配套工程以及4个农村供水工程建设进入扫尾阶段，完成投资11.62亿元，占年度计划的142%，累计完成投资19.82亿元，占概算投资的109%。投资8316万元，实施林业重点工程23.8万亩，天定、平定高速公路生态长廊建设完成年度任务。投资1.2亿元，实施土地开发整理项目29个，新增耕地1.29万亩，实现了耕地当年占补平衡。投资2.4亿元，新修梯田37.2万亩、综合治理水土流失面积526平方公里。安定区国家水土保持生态文明区（县）通过水利部评审，渭源县被列为国家级生态文明示范工程试点。投资8551万元，实施农村环境连片整治项目97个，建成农村户用沼气5680户。投资3.65亿元，实施以工代赈和易地扶贫搬迁项目95个、整村整乡推进及省级连片开发试点项目76个，减少贫困人口11.58万人。六盘山片区区域发展与扶贫攻坚启动会在定西召开，新一轮扶贫攻坚全面展开。

　　（六）致力社会和谐稳定，提高民生保障水平。坚持以改善民生为根本、和谐稳定为基础，积极推进基本公共服务均等化，全市财政投入民生和社会事业发展资金102.4亿元，增长28.2%。为民办理的10件实事全面完成。投资5.32亿元，改造建设中小学和幼儿园校舍29.2万平方米。积极推进普通高中新课程实验改革，努力提高教育教学质量，高考二本上线9226人，录取率23%。设立定西市贫困大学生救助资金，对2012年录取的200名贫困大学生进行了救助。加大力度实施校车安全工程，为1.12万名农村寄宿学生提供了校车服务。组织实施农村义务教育学生营养改善计划，为26万名学生提供了营养餐服务。深入推进医药卫生体制改革，大力发展中医药事业，城乡居民医疗服务保障水平不断提高。6所县级医疗机构、6县卫生监督所及医疗急救体系基础建设进展顺利，28个乡镇卫生院改扩建、89个标准化村卫生室建设任务圆满完成。发放小额担保贷款6.45亿元，新增城镇就业1.63万人，带动就业3.2万人。多渠道安置高校毕业生5554人。城镇职工各项社会保险稳健运行，城乡居民社会养老保险全面实施。城市低保、农村低保和农村五保人均供养标准分别提高15.5%、35.8%和14%，保障对象分别达到5.82万人、50.95万人和1.37万人。筹集财政资金2.61亿元，进一步提高了县聘代课教师、社区工作人员、文化计生专干、环卫工人、协警等低收入群体收入水平，对3.84万户城市低保户和农村五保户实行普惠制慰问，为行政事业单位离退休人员和城乡85岁以上老年人发放高龄补贴，提高干部职工和市区特困居民冬季取暖补贴，干部职工津补贴达到省直机关对应职级标准。为14943户城市住房困难家庭发放廉租住房租赁补贴4849万元，新开工保障性安居工程6958套，主体竣工6339套，改造农村危房3万户。扎实推进“联村联户、为民富民”行动，积极帮助贫困村、特困户改善生产生活条件，发展富民产业，加快脱贫致富进程。实施广播电视“村村通”工程10.9万户、“户户通”工程39万户，105个乡镇文化站、313家农家书屋全面建成，行政村农家书屋实现全覆盖。市级体育运动中心开工建设，体育健身工程向村社延伸。全面落实计划生育优先优惠政策，低生育水平持续稳定。建立安全生产全委会制度，深入开展专项治理行动，不断靠实政府属地管理、部门日常监管和企业主体责任，安全生产形势稳定好转。加强和改进食品药品检测监管，确保了群众饮食用药安全。深入排查化解各类社会矛盾，及时解决群众来信来访反映的困难和问题，积极回应社会关切。健全和完善应急管理体系，组织开展应急救援综合演练，努力提高突发公共事件应急处置能力。坚持关口前移、人员下沉，强化社会管理综合治理，依法严厉打击各类刑事犯罪，保持了社会和谐稳定。民族宗教、统计、科技、物价、市场监管、质量监督、气象、外事侨务、民兵预备役等工作和妇女儿童、老年人、青少年、残疾人、社会慈善等事业都有了新发展。

　　（七）致力优化发展环境，推进体制机制创新。不断加强制度建设，依靠制度创新工作。深入推进“效能风暴”和“效能问责”行动，问责296人。建立健全考核评价、督办反馈、行政问责制度，大力推行一线工作法、排位工作法、倒逼工作法，推动政风行风不断好转。建立完善主要领导定期赴省进京汇报争取项目、带头走出去招商引资及市直部门“一下一上”衔接项目等工作制度，深入开展“建设工程质量安全年”、“项目建设落地年”和“监督检查跟进年”活动，促进政府部门积极争取引进并加快实施项目。认真贯彻市人大常委会关于加强城市管理的决定，研究制定市区供热、供水、物业、交通等管理办法26个，筹资购置巡逻设备，实施“天眼”和24小时警灯闪烁工程，推动城区管理和治安巡逻步入常态化，并督促各县加强制度建设，强化相对集中行政处罚权工作，重点整治公路沿线集镇市场、交通秩序混乱问题，群众安全感和满意度进一步提高。文化体制改革基本完成，农村综合改革、集体林权制度综合配套改革、投融资体制改革和行政审批制度改革深入推进。定期向市人大及其常委会报告工作，主动加强与市政协联系，106件市人大代表建议和144件市政协委员提案全部办结。

　　（八）致力加强自身建设，提高依法行政水平。修订完善政府工作规则，严格工作程序，促进市政府班子成员在职权范围内独立地科学民主依法决策，积极主动抓好分管工作。建立市长务虚会议制度，着重就经济社会发展的重大问题进行深入研讨，不断理清工作思路，强化发展举措。完善市政府常务会议会前学法制度，专题学习了行政强制法、安全生产法、城乡规划法、统计法等13部法律法规，依法行政水平不断提高。落实市长碰头会议制度，及时沟通情况、部署工作、靠实责任，强力推动各项任务落实。加强政府信息公开，积极推行电子政务，建成电子民生和农村信息公开平台，政务服务能力不断提高。深入推进政风行风建设和民主评议工作，强化行政监察和审计监督。坚持“一岗双责”，认真落实党风廉政建设责任制，积极推进惩治和预防腐败体系建设，严格执行廉政准则，政府廉政建设不断加强。

　　回顾2012年工作，我们不会忘记，“5·10”岷县特大冰雹山洪泥石流灾害发生后，在党中央、国务院的亲切关怀下，在省委、省政府的坚强领导下，在国家有关部委、省直相关厅局和社会各界的帮助支持下，全市广大干部群众和灾区人民万众一心、众志成城，夺取了抢险救灾的重大胜利，灾后恢复重建有序有力有效推进。我们深切感到，全市经济社会发展取得的一切成绩，是市委正确决策、坚强领导的结果，是市人大、市政协监督支持的结果，是各级干部和广大群众团结奋斗、顽强拼搏的结果。在此，我代表市人民政府，向全市各族人民，向各民主党派、工商联、无党派人士、人民团体、离退休老同志，向驻定单位和人民解放军、武警部队官兵、公安民警，向所有关心支持定西发展的各界人士，表示衷心的感谢和崇高的敬意！

　　各位代表！当前，定西正处在后发赶超的关键时期，我们清醒地认识到，定西与全省全国同步建成全面小康社会还有很大差距，全市经济社会发展还存在不少困难。一是经济总量不足、产业结构不优、发展质量不高，工业化、信息化、城镇化和农业现代化水平低，调整经济结构、转变发展方式任务艰巨。二是城乡基础设施历史欠账较多，生态环境还需持续改善，支撑长远发展的大项目、引领产业优化升级的生产性项目还比较少，转型跨越发展的后劲有待进一步增强。三是城乡居民收入水平不高，贫困人口多，全面小康实现程度低，自然灾害频繁，扶贫攻坚任务繁重。四是地方财政困难，基本公共服务和社会保障能力弱，社会管理需要不断创新加强。五是对外开放程度低，金融服务不够完善，招商引资难度较大，资金瓶颈制约突出，节能减排压力加大，安全生产形势严峻。六是发展环境有待进一步优化，一些部门和工作人员思想解放不够，创新意识不强，工作作风不实，行政效能不高。对此，我们要高度重视，以务实的态度、扎实的举措，逐步加以解决。

　　2013年主要任务

　　2013年，是全面贯彻落实党的十八大精神的开局之年，是实施“十二五”规划的关键之年，也是为全面建成小康社会奠定基础的重要一年。综合分析判断，全市经济社会发展仍然面临诸多良好机遇和有利条件。从政策机遇看，随着新一轮西部大开发战略的深入推进，六盘山片区区域发展与扶贫攻坚规划的启动实施，国家和省上支持定西发展政策的逐步落实，我市加快转型跨越发展面临更多的政策叠加机遇。从发展条件看，随着兰渝铁路、宝兰客运专线、临渭高速等重大项目的加快建设，引洮供水一期工程的即将建成，我市加快转型跨越发展具备了更加坚实的基础条件。从产业支撑看，随着经济开发区和工业集中区承载功能的逐年增强，马铃薯、中医药、畜草和铝产业等特色优势产业发展水平的不断提升，我市加快转型跨越发展的产业优势更为突出。从内部环境看，干部群众期盼发展的愿望强烈，并随着改革开放的不断深入，体制机制的创新完善，社会管理的持续加强，干部作风的不断改进，我市加快转型跨越发展的氛围更加浓厚。站在新的起点上，我们必须准确把握发展趋势，用足用好各项政策，充分发挥比较优势，积极运用宝贵经验，自我加压，主动作为，奋力推动定西经济社会加快转型跨越发展。

　　政府工作的总体要求是：全面贯彻落实党的十八大和省第十二次党代会、市第三次党代会精神，以科学发展为主题，以转变发展方式为主线，以推进新型工业化、社会信息化、特色城镇化、农业现代化为战略基点，坚持兴农强工、做大城镇、扩充总量、提升水平，精心打造中国薯都、中国药都和甘肃畜草产业大市、陇中特色文化大市，努力实现项目工作、产业开发、工业发展、城镇建设、文化繁荣、生态文明、民生改善新突破，为全面建成小康社会奠定坚实基础。

　　经济社会发展的主要预期目标是：生产总值增长13.5%以上，固定资产投资增长40%以上，规模以上工业增加值增长22%，社会消费品零售总额增长18%以上，大口径财政收入、公共财政预算收入均增长20%，城镇居民人均可支配收入、农民人均纯收入均增长18%。居民消费价格涨幅控制在3.5%左右，人口自然增长率控制在6.9‰以内，城镇登记失业率控制在4%以内，节能减排控制在省下达指标之内。

　　（一）深入实施项目带动战略。按照省委、省政府实施“3341”项目工程的战略部署，全力推进项目建设，确保完成固定资产投资553亿元以上，拉动经济较快增长。围绕国家宏观调控政策、产业扶持导向及市场需求变化，筹措前期经费6000万元以上，编制沿洮扶贫开发等发展规划，认真做好项目前期工作，谋划千万元以上项目1200个以上，不断充实完善项目库。争取渭河源区生态保护与综合治理规划通过国家批复并组织实施。进一步完善项目争取机制，强化与国家有关部委和省直厅局有效衔接，争取落实政策性资金70亿元以上。重点抓好全省观摩大项目和领导责任制项目，实施500万元以上项目1500个，其中亿元以上项目180个。全力抓好征地、拆迁、安置等协调服务工作，确保宝兰客运专线、华能华家岭风电二期、750千伏输变电、临渭高速、兰州至定西输气管道等省部属重大项目顺利推进。强化各级责任，加快引洮一期定西支渠及配套工程扫尾建设，组织开展工程质量“回头看”，全面排查和消除工程质量隐患，及早做好通水调试、水资源科学高效利用及运行管理工作，确保按期发挥效益。全面落实招商引资优惠政策，扩大对外开放，重点瞄准世界500强、全国500强企业，依托园区和特色优势产业，采取节会招商、产业招商、小分队招商等多种形式，力争年内引进千万元以上项目150个，到位资金增长40%以上。跟踪落实灾后恢复重建资金，加快规划实施，确保教育项目8月底前投入使用，卫生、计生等项目9月底前全面竣工，其他项目完成年度任务。进一步强化项目建设的质量、资金和施工安全监管，不断提升项目建设和管理水平，确保工程质量和效益。

　　（二）加快推进农业现代化。着眼促进农业增效、农民增收，全面提升特色优势产业，积极培育富民多元产业，做大做强区域首位产业，引导扶持农民专业合作社、龙头企业、种养殖大户通过土地流转发展集约经营，努力提升农业现代化水平。围绕打造“中国薯都”，着力加强良种繁育、标准化种植、精深加工和科技研发工作，推动原原种生产能力达到6亿粒，建立标准化种植基地250万亩。全力抓好圣大方舟生物质基材、清吉公司无矾方便粉丝、爱兰薯业和金大地公司速冻薯制品、蓝天公司淀粉糖及壁纸胶生产线、富民生态、国丰科技等龙头企业重点项目，推动马铃薯产业开发向全产业链、循环化发展。加快组建马铃薯工程研究院、种业协会和综合检测中心，不断提升马铃薯产业研发水平。马铃薯产业总产值达到105亿元。围绕“中国药都”建设，突出抓好标准化种植、现代制药、仓储物流和科技创新工作，积极引进试验设施种植名贵中药材。建立种子种苗繁育基地5万亩、标准化种植基地55万亩。新建中药提取物生产线5条，中药饮片及提取物加工能力达到16万吨。争取组建中医药养生保健研究院，筹建甘肃岷县当归研究院。中医药产业总产值达到260亿元。围绕建设甘肃畜草产业大市，充分发挥玉米秸秆、优质多年生牧草和天然草场等资源优势，坚持农畜草结合、种养加并举，推动农畜循环发展。投入财政扶持资金1亿元，青贮氨化玉米秸秆200万吨，新建规模养殖场250个以上、规模养殖户1万户以上，加快建设广德福丰公司特色养殖富民产业园、天耀草业草畜肥循环农业示范等重点项目。培育引进畜草产业加工龙头企业，不断提升畜草产业发展水平。畜草产业总产值达到80亿元。围绕引洮水资源高效利用，加快发展蔬菜产业和小杂粮、林果、花卉等富民多元产业，新建日光温室1000座、塑料大棚1.6万亩，蔬菜种植面积达到60万亩，总产值提高到60亿元。大力发展农业机械化，强化农机农艺融合配套，完成全膜双垄沟播种植350万亩以上。

　　（三）大力发展新型工业。围绕壮大工业经济总量，加快实施工业发展“倍增计划”，着力打造千亿元产业、千亿元园区和十亿元企业，加快培育中小企业梯队和小微企业集群，支持陇西县率先向工业强县迈进。投入财政资金2.1亿元，重点支持4个经济开发区和3个工业集中区加快扩容升级，新引进入驻企业50户以上，力争实现产值40亿元以上。启动建设定西经济开发区循环经济产业园商住商服区和定西食品工业园，积极推进陇西经济开发区循环化改造试点。深入推进工业发展十大行动计划，实施千万元以上工业项目135个，推动马铃薯精深加工、中医药、铝冶炼及深加工、矿产建材、电能及新能源、精盐化工和装备制造等七大主导产业做大做强。全力做好协调服务，促进陇西现代中药及保健食品产业园、中国药材公司岷县产业园等重大项目尽早开工，甘肃招金贵金属冶炼、祁连山水泥二期、中盛铝业铝合金建筑型材等重大项目完成年度投资任务，并大力发展风电、生物质能等可再生能源和新能源，积极培育生物医药、低碳环保节能建材等战略新兴产业，力争完成工业固定资产投资133亿元。加大承接产业转移力度，力争到位资金74亿元以上。促进科技创新企业和小微企业健康发展，力争培育规模以上工业企业30户。大力推进质量振兴，充分发挥“扶正”等中国驰名商标效应，争创甘肃名牌产品5个以上。加强预警监测监控，全面完成节能减排任务。认真落实各项扶持优惠政策，促进民营经济和实体经济加快发展。

　　（四）推动服务业转型发展。适应市场和消费需求，着力改造提升传统服务业，大力发展现代服务业，推动生产性服务业与生活性服务业协调发展。围绕建设陇中特色文化大市，加强文化基础设施建设，全面建成市博览中心、文化艺术中心，启动建设数字图书馆、电子阅览室。全力打造文化产业聚集发展平台，加快建设临洮马家窑、陇西李氏、岷县洮砚、通渭书画等文化产业园。依托园区引进一批文化龙头企业，扶持现有文化企业发展壮大，认定一批市级文化产业重点企业。加快贵清山、渭河源等景区基础设施建设，深度开发临洮洮之苑文化休闲街区、洮河漂流、温泉疗养、中药养生、盐疗漂浮等旅游休闲项目，加快实施鸣柳龙源镇、卧龙湾休闲水镇等重点项目。加强区域协作，打造精品旅游线路，加快开发富有地域和文化特色的旅游产品。新发展农家乐200户以上，旅游综合收入增长20%以上。不断创新完善金融服务体系，力争甘肃银行在定西设立分行，农业发展银行在临洮设立支行，兰州银行在定西设立2个县级支行，建设银行通渭支行挂牌运营，争取将安定区农村信用社改制为定西农村商业银行。继续扶持发展小额贷款公司和担保公司，支持各类金融机构在农村设立金融综合服务室和便民服务站，创新农村金融产品，推进信用体系建设。加强银政企合作，充实中小企业融资需求项目储备。推进市级融资平台建设，加快企业上市融资，力争陇原中天公司成功上市。进一步争取农业部和省政府支持，加快建设国家级定西马铃薯批发市场，推动“中国薯都”在构建全国马铃薯物流集散中心、价格形成中心、信息传播中心、科技交流中心和会展贸易中心等方面取得重要进展。加强“定西马铃薯”中国驰名商标宣传、使用和管理，不断扩大品牌效应。加快康美药业等现代物流园建设进度，力争引进培育大型物流企业3户以上，新建和提升改造农贸市场10个。认真落实支持服务业发展各项政策，促进生产性服务业增加值增长20%以上，服务业增加值增长14%左右。

　　（五）统筹推进城乡基础建设。全面构建以城带乡、城乡互动发展新机制，力争城镇化率提高到28%以上。完善市县城镇控制性详规和专项规划，注重重点区段和主要节点的城市设计，不断提升城镇规划水平。围绕“五宜”城市建设，市区实施项目72个，完成投资42亿元以上。新城区重点启动亚行贷款道路等基础设施项目，加快实施中和教育港、体育运动场馆等公共设施项目，确保新城区迁建单位顺利搬迁，配套完善餐饮、购物、金融等综合服务体系。旧城区重点实施西岩新村改造、中华路中段城市综合体建设等项目，着力营造宜居宜商环境。各县城及重点小城镇建设完成投资50亿元以上，进一步增强辐射带动功能。完成通渭马营至陇西云田二级公路等项目部分路基工程，抓好定临公路安定过境段建设，加快建设通乡通村公路，争取渭源至武都高速，通渭至榜罗、安定巉口至会宁郭城二级公路，国道212线境内改造早日开工，完成公路投资18亿元。推进中小河流治理、规模节水示范、山洪灾害防治等重点水利工程，新增节水及有效灌溉面积3.5万亩。全面实施六盘山片区区域发展与扶贫攻坚规划，抓好60个整村推进项目，易地扶贫搬迁贫困人口1.6万人，减少贫困人口10万人以上。结合新农村建设，深入开展“清洁家园、美丽乡村”创建活动，强化交通干道沿线及重点小城镇交通秩序、市场秩序和环境卫生综合整治，营造管理有序、特色鲜明、村容整洁的生产生活环境。

　　（六）切实加强生态文明建设。树立尊重自然、顺应自然、保护自然的生态文明理念，推进绿色发展、循环发展、低碳发展，加快建设资源节约型、环境友好型社会。围绕消灭荒山、绿化定西总目标，编制生态文明建设规划，积极争取国家批准设立生态建设、保护与补偿实验区。投入财政资金5000万元，争取和整合相关项目资金，千方百计加大绿化建设投入，加快实施林业发展规划、林业特色产业发展规划和绿色长廊建设规划，大力推进退耕还林、三北防护林等重点生态工程，深入开展全民义务植树，加快城市面山绿化及文冠果等经济林建设。严把苗木关，加强林木管护，努力提高成活率，完成造林绿化22万亩，森林覆盖率达到13.7%。实施黄土高原水土保持示范区规划，新修梯田30万亩，综合治理水土流失面积350平方公里。强化“村村响”等山洪灾害防治县级非工程措施，建立健全预报预警机制，切实提高自然灾害防御能力。把资源消耗、环境损害、生态效益纳入经济社会发展评价体系，全面落实投资项目能评环评准入制度，大力争创国家级和省级生态乡镇。加强土地开发整理、矿产资源管理和基本农田保护，积极推进集约节约用地，保障重点建设项目和民生工程用地。

　　（七）积极推进重点领域改革。继续推进土地流转，深化集体林权制度综合配套改革，探索农村道路、小型水利管护方式，逐步建立农村公益性项目农民自管新机制。适应城镇化发展新要求，建立覆盖县城和重点小城镇的管理制度体系，加强和改进行政管理综合执法，不断提高城镇管理水平。抓好财政支出绩效考评制度试点，建立健全“三公”经费及政府、部门预算公开机制，全面推行公务卡支付制度，增强公共财政公开透明度。加快统计方法制度改革，严格依法科学统计，完善统计服务功能，确保统计数据真实可靠。全面推进农村金融服务综合改革试点，积极推行农村“三权”抵押贷款。进一步完善金融服务经济发展评价激励办法，促进金融机构不断提升服务水平。继续深化医药卫生体制改革，加大公立医院和基层医疗机构改革力度，大力推行绩效管理。开展大病医疗保险试点，建立大病医疗保险和救助机制，健全城乡居民医保体系，完善突发公共卫生事件应急和重大疾病防控机制。整合公共服务职能，推进市县政府政务服务中心规范化、标准化建设，规划建设公共资源交易中心和电子监察系统，完善电子民生平台运行。精简下放一批行政审批事项，建立健全公开透明、规范高效的行政运行体制。

　　（八）着力保障和改善民生。认真落实教育优先发展战略，高度重视学校安全，狠抓校长、教师队伍建设，积极争取实施农村初中校舍改建、薄弱学校改造、教师周转宿舍等建设项目。优化教育资源配置，合理布局并加快建设城区学校，大力发展学前教育，促进义务教育均衡发展。支持职业教育、高等教育、特殊教育和民办教育健康发展，抓好校车安全、营养改善、困难学生资助，强化寄宿制学校管理，努力办好人民满意的教育。进一步健全公共卫生服务体系，大力实施基层中医药服务能力提升工程，积极争创全省中医药工作先进市。实施好妇幼保健站、中医院、乡镇卫生院辅助用房等建设项目。科学整合市区医疗卫生资源，全面完成市医院搬迁。落实更加积极的就业政策，推进大学生创业园和见习基地建设，发挥创业带动就业倍增效应，新增城镇就业1.71万人。制定农民收入、城镇居民可支配收入“倍增计划”，促进城乡居民收入持续较快增长。加强职业技能培训，输转剩余劳动力65万人次，创劳务收入76亿元。加快社会保障体系建设，强化城乡居民社会养老保险和城乡居民最低生活保障，健全完善社会救助制度，做好失地农民保障工作。实施科技创新工程，开展民生科技试点，推动科技成果尽快应用于生产领域。提高广播电视节目质量，加快无线发射台站建设，实现“户户通”工程地面数字节目全覆盖。广泛开展全民健身运动，促进群众体育和竞技体育全面发展。坚持统筹解决人口问题，积极实施利益引导，全面提供优质服务，增强家庭发展能力，继续稳定低生育水平。高度重视安全生产和食品药品监管，不断总结经验教训，建立长效工作机制。深入开展矛盾纠纷排查化解活动，妥善解决群众合理诉求。抓好社会管理创新综合试点，建立健全社会管理综合治理协调机制和重大决策社会稳定风险评估机制，依法严厉打击各类刑事犯罪，全力维护社会和谐稳定。保障妇女儿童合法权益，健全残疾人社会保障和服务体系。加强国防后备力量建设，做好征兵、双拥优抚、转业退伍军人安置工作。加强粮食购销、市场监管、民族宗教、外事侨务、地震、人防、气象、防灾减灾、公共机构节能等工作，推动地方志、红十字会、老年人、青少年、社会慈善等事业健康发展。

　　解民忧、促民富、保民安是各级政府的重要职责。今年，在认真办好省政府实事的同时，多渠道筹集资金9.45亿元，继续为民办好10件实事：（1）改善46所农村薄弱学校办学条件，新建校舍2.17万平方米；建成幼儿园14所，新建校舍2.18万平方米；加快安定区福台中学等4所学校教学实验楼建设，新建校舍2.13万平方米。（2）为500名贫困白内障患者实施复明手术；为1.1万对计划怀孕夫妇实行孕前优生健康检查。（3）改扩建乡镇卫生院20所。（4）改造农村危房1.8万户；新建农村户用沼气2640户。（5）建成通村公路100条、550公里，全市行政村通水泥或沥青路率达到50%以上。（6）建成18个乡镇社区体育健身中心；为困难群众办理法律援助案件500件。（7）新增小额担保贷款3亿元，支持6000人自主创业，带动就业1.2万人以上。（8）将100个贫困行政村列入互助资金试点村，积极探索扶贫开发新模式。（9）实施农村安全饮水工程，解决25万人饮水不安全问题。（10）发放住房租赁补贴8859户，新建各类保障性住房3251套。加强政府自身建设　　面对转型跨越发展的新形势、新任务，面对全市人民群众过上幸福美好生活的新期待、新要求，我们一定要加强自身建设，努力建设学习型、服务型、创新型政府。

　　（一）提高科学决策水平。加强学习，不断提高理论和法规政策水平。围绕事关全市发展的重大问题，深入开展调查研究，坚持问政于民、问需于民、问计于民，从基层和群众的新鲜实践中汲取智慧和力量。健全完善公众参与、专家论证、风险评估、社会公示、合法性审查等决策制度，严格遵守政府工作规则，认真执行民主集中制，重大问题及时向市委请示，不断提高科学决策水平。

　　（二）规范依法行政行为。坚持把依法行政贯穿于政府决策、执行监督全过程，严格按法规和程序履行职责，增强有权必有责、用权受监督意识，自觉接受市人大依法监督和市政协民主监督，高度重视舆论监督和社会公众监督，把各项工作纳入规范化、法制化轨道。全面推进行政执法责任制，加大行政执法监督力度，确保秉公执法、严格执法和文明执法，不断提高政府依法行政水平。健全行政复议、信访申诉等制度，切实维护群众利益。

　　（三）不断改进工作作风。认真落实中央政治局关于改进工作作风密切联系群众的“八项规定”、省委常委会“十项规定”和市委常委会有关规定，严格控制压缩会议，大力精简文件简报，切实减少评比检查活动，下决心改进会风文风。讲真话、报实情、干实事，不断改进工作作风，提高工作实效。深入开展“双联”行动，着力整治庸懒散奢等不良风气，坚决克服形式主义、官僚主义，以扎实的工作作风，推动各项任务全面落实。

　　（四）严肃行政效能问责。深入开展“政策规划落实年”、“管理服务创新年”活动。健全完善重点工作跟踪落实、督办问责、考核评价制度，严肃查处慢作为、不作为、乱作为等失职渎职行为，坚决杜绝推诿扯皮、敷衍塞责、吃拿卡要，确保政令畅通。继续推进“效能风暴”、“效能问责”行动，促进政府各部门和各级干部增强责任意识、效能意识，聚精会神谋发展，集中精力抓落实。

　　（五）着力加强廉政建设。坚持从严治政，严格执行党风廉政建设责任制，全面推进惩治和预防腐败体系建设。加强行政监察和审计监督，强化工程建设、国有资产、土地出让、政府采购等重要领域监管，坚决纠正损害群众利益的不正之风，营造风清气正、廉洁高效的政务环境。严格执行廉政准则，严格遵守住房、公务出行、车辆配备等有关规定，严格控制和压缩“三公”支出，进一步规范公务接待，降低行政成本，树立为民务实清廉的良好政府形象。

　　各位代表！做好今年各项工作，任务艰巨，责任重大，使命光荣。我们一定在党的十八大精神指引下，在省委、省政府和市委的坚强领导下，在市人大、市政协的监督支持下，解放思想、锐意进取，求真务实、埋头苦干，为推动全市转型跨越发展、建设开放文明和谐新定西作出新的贡献！
